# Mental training driven by a winning mindset in the Chinese national ice hockey team: implications of medal-winning practices for the youth Ice hockey training system

**DOI:** 10.3389/fspor.2026.1649235

**Published:** 2026-04-30

**Authors:** Zhiwen Sun, Zhigang Song, Yongmei Hu, Rui Gao, Yanhua Guo, Xiaoting Sun, Luning Wang, Yue Hu, Meng Zhang, Na Zhang

**Affiliations:** 1 School of Education & Psychology, Tianjin University of Sport, Tianjin, China; 2 China Volleyball College, Beijing Sport University, Beijing, China; 3 Faculty of Education, University of Macau, Macao SAR, China; 4 Teaching and Research Office of Physical Education, Tianjin Hexi District Huaxing School, Tianjin, China

**Keywords:** mental training, psychological capacities, psychological model of ice hockey success, women's 3v3 ice hockey, youth training program

## Abstract

**Background:**

Based on Miller's psychological model of ice hockey success, this study aims to develop a specialized and systematic mental training program for Chinese female 3v3 ice hockey athletes, providing empirical evidence for improving the competitive performance of China's ice hockey teams and the psychological development of reserve talents.

**Methods:**

Twenty-two athletes from the Chinese National Women's 3v3 Ice Hockey Team were recruited as participants. According to the six core dimensions of the psychological model of ice hockey success, a systematic mental intervention program was constructed, including goal orientation, emotional regulation, positive self-talk, teamwork, and other modules.The Maslach Burnout Inventory-General Survey (MBI-GS), Perceived Stress Scale (PSS), and competitive performance indicators were used to assess outcomes before and after the intervention. Paired-sample t-tests and repeated-measures ANOVA were applied to examine differences across measurement points.

**Results:**

After the intervention, athletes’ occupational burnout decreased significantly.Scores for emotional exhaustion, cynicism, and reduced professional efficacy dropped from 4.23 ± 1.25, 3.87 ± 1.12, 4.01 ± 1.18 to 3.12 ± 1.08, 2.95 ± 0.98, 3.05 ± 1.02, respectively.Paired-sample t-tests revealed highly significant differences for all subscales (t = 5.31–5.81, all *p* < 0.001). Highly significant improvements were also observed in perceived stress, decision-making accuracy under high pressure, tactical execution efficiency, and other core competitive indicators.Group×time interaction effects for perceived stress and decision-making accuracy were both highly significant (all *p* < 0.001).

**Conclusion:**

The systematic mental training system based on Miller's psychological model of ice hockey success can significantly improve the mental health and performance-related psychological capacities of female 3v3 ice hockey athletes.Verified in the preparation practice of the national team, this program provides a replicable framework for mental training in youth ice hockey athletes, with core techniques including goal decomposition, mindful breathing, and positive self-talk.

## 1 Introduction

Ice hockey is a team sport characterized by high-speed skating, intense physical contact, and sophisticated tactical coordination (1), which imposes extremely high demands on athletes’ physical fitness, technical and tactical proficiency, and mental resilience (2). Compared with traditional ice hockey, 3v3 ice hockey features faster offensive-defensive transitions, more frequent decision-making per unit time, and denser physical contact. This implies that female 3v3 ice hockey athletes endure greater mental load during competition, and their on-ice performance is highly associated with psychological abilities such as emotional regulation, attention maintenance, and cognitive decision-making (3). In recent years, with the global popularization of winter sports, the competitive level of women's ice hockey has improved rapidly. Mental skills have become one of the core factors determining the upper limit of competitive performance among elite female ice hockey athletes (4). As a core psychological resource for female athletes to cope with high competition pressure and adapt to high-intensity confrontation, psychological resilience is even a key trait that determines the stability of their athletic performance and their career lifespan (5).

The Psychology of Ice Hockey Success Model was proposed by Miller, based on the practical experience of hundreds of elite ice hockey athletes and coaches worldwide. The model identifies six core psychological dimensions underlying ice hockey performance: goal setting, emotional management, positive imagery, positive self-talk, hard training and physical condition, and a winning mindset, providing a systematic theoretical framework for mental skills training in ice hockey (6). This model has been widely adopted by elite ice hockey teams across North America and Europe. Existing research has confirmed that psychological interventions based on this model can effectively improve ice hockey athletes’ emotional regulation, on-ice decision-making efficiency, and competitive performance stability. Meanwhile, a large body of research in international sport psychology has verified that mental skills training—including mindfulness training, positive self-talk, and goal-oriented interventions—can significantly enhance elite female athletes’ mental resilience, stress coping ability, and competitive performance (7). Mindfulness training effectively reduces competitive anxiety and improves attention control and emotional regulation among female athletes (8). The work of Van Raalte et al. (8) indicates that targeted positive self-talk interventions significantly improve female athletes’ self-efficacy and reduce negative thinking in competition. A longitudinal study by Kavoura et al. (9) on elite female athletes found that systematic mental resilience training significantly decreases athletes’ burnout and enhances the stability of their long-term competitive performance (10). Recent studies have further confirmed that psychological skills such as mindfulness, positive self-talk, and goal setting can exert a sustained positive effect on athletic performance by fostering athletes’ psychological resilience (11). Meta-analyses have verified the stable promoting effect of positive self-talk on athletic performance, and psychological skill training combining self-compassion with positive self-talk can more effectively help female athletes cope with gendered stress and self-critical thinking in the competitive sports environment, further enhancing the positive effect of psychological intervention on mental health and on-court performance (12, 13).

Although some progress has been made in relevant research on psychological intervention in ice hockey, existing studies still have three core deficiencies: first, most existing studies focus on psychological intervention for traditional male ice hockey players (14), and there is an extreme lack of specialized psychological training research on women's 3v3 ice hockey, an emerging Olympic discipline. No systematic psychological intervention program adapted to the characteristics of women's 3v3 ice hockey has been formed yet. However, specialized studies on elite female athletes have clearly shown that psychological resilience intervention must be closely combined with the specialized characteristics of the discipline and the psychological needs of female athletes to achieve the maximum intervention effect (5). Second, most existing psychological studies on female ice hockey athletes mainly focus on passive remedial interventions such as post-competition psychological counseling and anxiety relief, lacking the construction of preventive and full-cycle psychological training systems based on theoretical models. At the same time, there is little attention paid to the impact of psychological intervention on long-term development indicators such as athletes’ burnout and team collaboration. Although meta-analyses have confirmed the positive effect of mindfulness training on the psychological state and psychological resilience of elite athletes, and classic theories have clarified the key value of psychological resilience for the long-term competitive career of elite athletes, existing studies still lack systematic preventive intervention programs that combine these evidence-based conclusions with the specialized scenarios of women's ice hockey (15). Third, most existing domestic studies on ice hockey psychological training are either theoretical discussions or short-term interventions of a single psychological module, lacking systematic empirical research that combines mature theoretical models with the preparation practice of Chinese female ice hockey athletes. At the same time, no replicable psychological training system that can be extended to the cultivation of adolescent reserve talents has been formed (16).

Against the above research background and existing limitations, this study takes athletes from the Chinese National Women's 3v3 Ice Hockey Team and athletes of various age groups in the National Ice Hockey Youth Training Camp as research participants (17). A systematic multi-module psychological training system is constructed based on the Ice Hockey Success Psychology Model, and the intervention effect of this system is verified through empirical research (1). This study proposes the following core research hypotheses: (1) Systematic psychological intervention based on the Ice Hockey Success Psychology Model can significantly reduce the level of occupational burnout, and improve perceived stress, emotion regulation ability, and attention maintenance ability among Chinese women's 3v3 ice hockey athletes (18). (2) This psychological training system can significantly improve performance-related indicators such as decision-making accuracy under high pressure and team tactical execution efficiency (19). (3) The graded psychological training system constructed based on this model is compatible with the developmental characteristics of adolescent ice hockey athletes and is replicable for promotion in the youth talent development system. This study aims to provide empirical support for the psychological training of Chinese women's 3v3 ice hockey athletes in competition preparation, and to offer feasible practical schemes for cultivating psychological resilience in adolescent reserve talents for China's ice hockey program (20).

## 2 Materials and methods

### 2.1 Participants

The study included 22 athletes from the Chinese National Women's 3v3 Ice Hockey Team. All participants were volunteers who provided written informed consent. Ethical approval for this study was obtained from the ethical review committee in a previous study by the first author, with approval number TJUS-2025-086.

### 2.2 Experimental procedures

#### 2.2.1 Goal-Oriented psychological training

The intervention was implemented during the national team training camp over a 4 week period. One specialized psychological training session was conducted per week, for a total of 4 sessions. Each session lasted 30 min, including instruction, practical operation, and immediate feedback. All sessions were scheduled on rest days to ensure athletes were in good physical and mental condition and to avoid interference with regular training and recovery (21). After signing informed consent and a confidentiality agreement, participants completed standardized tests in a quiet team meeting room, where all intervention sessions were also delivered. Training content included Clarification of personal motivation, Breakdown of preparation goals, Integration of individual and team goals and Development of psychological contingency plans for high-pressure competition scenarios (22).

#### 2.2.2 Emotion regulation training

The intervention was conducted during the national team training camp over an 8 week period. One specialized psychological training session was held per week, for a total of 8 sessions. Each session lasted 30 min, including instruction, practice, and immediate feedback. All sessions were scheduled on rest days to ensure athletes’ well-being and avoid disruption to normal training (16). Training content was structured as follows: Weeks 1–2: awareness of personal emotions and basic “4-7-8 breathing” training; Weeks 3–4: mindfulness education and static mindfulness practice; Weeks 5–6: emotional journaling and three-level stress decomposition (emotion review, mindfulness or breathing relaxation, establishment of rapid emotion-regulation cues); and Weeks 7–8: construction and implementation of a programmed emotion regulation protocol. After providing informed consent and signing a confidentiality agreement, participants received the intervention and completed post-testing, with instructions not to discuss specific training content with other participants during the experiment. This study employed a 2 (group: experimental, control) × 2 (time: pre-training, post-training) mixed factorial design, where psychological indicators were measured using the Perceived Stress Scale and athletic performance indicators were assessed via athlete self-report (23).

#### 2.2.3 Positive self-talk training

The intervention was delivered during the national team training camp over a 4 week period. One specialized psychological training session was conducted per week, for a total of 4 sessions. Each session lasted 20 min, including instruction, practice, and individual and group feedback. All sessions were scheduled on rest days to maintain athletes’ physical and mental state without disturbing regular training (8). After signing informed consent and a confidentiality agreement, participants collectively learned the positive self-talk training materials (Table 1) and acquired the theoretical foundation of positive self-talk. Participants then completed writing self-talk templates for post-failure scenarios, conducting positive self-talk training following injury or discomfort, and developing team self-talk templates for post-competition setbacks (24).

**Table 1:** Examples of positive self-talk psychological training.

Match scene/mental state	Example of negative self-talk	Engage in positive self-motivating conversations
When you blame yourself for a mistake	“I screwed up again and it's gonna hurt the team.”	“Mistakes are the ladder of growth!Adjust immediately and I can seize the opportunity next time."
Under pressure from strong rivals	“They are too strong to win.”	“The greater the challenge, the more valuable the breakthrough!I have unique advantages,and every confrontation is a moment to prove myself!”
When your physical strength is low and you feel tried	“I can't hold on.I can't get up speed.”	“My potential is far beyond what it is now! Every slide is a transcendence of the limit,and I can control the pace of the race by gritting my teeth.”
When you’re behind and youer sprits are low	“Too bad, we’re losing this.”	“The game is not over until the last second!Our team can reverse the situation, I will try my best to overtake!”
Anxiety before the crucial ball	“If it fails,everyone will be disappointed.”	“This is the chance to show your strength!Focus on the action, believe in the results of training, I am born for the moment!”
Incompetenet in technical movements and lack of confidence	“I can't do this move right.”	“More practice is more progress! Every attempt strengthens muscle memory, I can definitely do it perfectly!”

#### 2.2.4 Team collaboration psychological training

The intervention was conducted during the national team training camp over a 4 week period. Three specialized psychological training sessions were held per week, totaling 12 interventions, with each session lasting 20 min (including instruction, practical operation, and individual and inter-group feedback). All sessions were scheduled on rest days to ensure athletes were in good physical and mental condition and to avoid interfering with their regular training and rest (25). After signing the informed consent form and the experiment confidentiality agreement, participants were instructed not to discuss the specific content of the training with non-participants. The training content was structured as follows: Week 1: team role cognition and responsibility consensus training; Week 2: establishment of team tactical trust and communication norms; Week 3: team goal integration and collaborative scenario simulation training; Week 4: development of team psychological contingency plans and solidification of collaborative models for high-pressure competition scenarios. A follow-up evaluation was conducted 1 month after the intervention, tracking the core indicators of team collaboration (role misalignment frequency, tactical execution completeness, and effective communication frequency among team members) during the 1-month follow-up period and 8 official matches. All the above core indicators were determined through athlete self-evaluation and coach evaluation (26).

### 2.3 Experimental materials

#### 2.3.1 Maslach burnout inventory-general survey (MBI-GS)

This study used the revised Maslach Burnout Inventory-General Survey (MBI-GS) for assessment. The scale consists of 15 items and measures burnout from three dimensions: emotional exhaustion, negative affect, and personal accomplishment. A 7-point Likert scale was adopted, where 0 indicates “never” and 6 indicates “every day”. Higher scores indicate a more severe level of burnout. According to the study by Li and Shi (27), the adjusted MBI-GS has a high consistency with the original structure. This confirms that the MBI-GS has good construct validity in China and can scientifically and effectively measure burnout, thereby providing a solid foundation for the reliability of the data in this study (10).

#### 2.3.2 Perceived stress scale

The Perceived Stress Scale, developed by Cohen et al., is an effective tool for assessing individuals’ perceived level of daily stress situations. This scale has good reliability and validity among Chinese adults. It consists of two dimensions: tension and loss of control, with a total of 14 items, including 6 positive items and 8 reverse-scored items. A 1–5 rating system was used for the scale, with a total score ranging from 0 to 70. The total score is positively correlated with individuals’ perceived stress level, that is, the higher the score, the more significant the participant's perceived stress. According to the score classification: 14–28 points indicate low perceived stress, 29–42 points indicate moderate perceived stress, 43–56 points indicate high perceived stress, and 57–70 points indicate extremely high perceived stress.

## 3 Results

### 3.1 Goal-Setting training

A paired-samples t-test was conducted to compare the scores of the Maslach Burnout Inventory (MBI) among 22 Chinese women's 3v3 ice hockey players before and after goal-setting training, with all 22 players’ results included in the analysis. The results showed (descriptive statistics are presented in Table 1): on the emotional exhaustion dimension, t(21) = 5.81, *p* < 0.001; on the cynicism dimension, t(21) = 5.31, *p* < 0.001; and on the reduced personal accomplishment dimension, t(21) = 5.31, *p* < 0.001. These results indicate that goal-setting training effectively improved the athletes’ ability to set psychological goals.

### 3.2 Emotion regulation training

A 2 (group: experimental group, control group) × 2 (time: pre-test, post-test) repeated measures analysis of variance (ANOVA) was conducted to compare the relevant psychological indicators of 22 Chinese women's 3v3 ice hockey players before and after emotion regulation training. All 22 players’ results were included in the analysis, with 11 players in each group, and the descriptive statistics are presented in Table 2.

**Table 2:** Descriptive statistics of the Job burnout scale (M ± SD).

Factor name	Pre-test	Post-test
Emotional exhaustion	4.23 ± 1.25	3.12 ± 1.08
Negativity and apathy	3.87 ± 1.12	2.95 ± 0.98
Low sense of accomplishment	4.01 ± 1.18	3.05 ± 1.02

Regarding the results of the psychological indicator (Perceived Stress Scale score) (descriptive statistics are presented in Table 3), the interaction effect between group and time was significant, F(1, 20) = 64.54, *p* < 0.001. Therefore, further simple effect analysis was performed. *post-hoc* test results showed that there was a significant difference before and after training in the experimental group, F(1, 20) = 148.19, *p* < 0.001; while there was no significant difference before and after training in the control group, F(1, 20) = 0.66, *p* = 0.43. These results indicate that emotion regulation training effectively alleviated the athletes’ perceived stress.

**Table 3:** Descriptive statistics of indicators related to emotion regulation training (M ± SD).

Indicator classification	Evaluation indicators	Experimental group (pre-training)	Experimental group (post-training)	Control group (pre-training)	Control group (post-training)
Psychological indicators	Perceived Stress Scale score	28.7 ± 3.5	19.2 ± 2.9	28.4 ± 3.3	27.8 ± 3.1
Physiological indicators	High pressure scene decision accuracy（%）	65.3 ± 4.8	82.6 ± 5.1	64.8 ± 4.6	66.2 ± 4.3
	Fatigue recovery time（minutes）	15.2 ± 2.3	9.8 ± 1.6	14.9 ± 2.1	14.5 ± 2.0

Regarding the results of the athletic performance indicator (decision-making accuracy in high-pressure scenarios), the interaction effect between group and time was significant, F(1, 20) = 23.02, *p* < 0.001. Therefore, further simple effect analysis was conducted. *post-hoc* test results showed that there was a significant difference before and after training in the experimental group, F(1, 20) = 53.74, *p* < 0.001; whereas there was no significant difference before and after training in the control group, F(1, 20) = 0.30, *p* = 0.59. These results suggest that athletes could improve their athletic performance by regulating their psychological state in stressful situations after training.

### 3.3 Positive self-talk training

During the positive self-talk training, athletes adopted more positive statements to cope with challenges in different scenarios. In subsequent training and competitions, findings from athlete interviews and coach interviews indicated that athletes were more positive than before participating in the training camp, and both individuals and the team were more positive and composed when facing challenges.

### 3.4 Team collaboration psychological training

A paired-samples t-test was conducted to compare the changes in on-ice performance of 22 Chinese women's 3v3 ice hockey players before and after team collaboration psychological training. The results of all 22 players in 8 matches were included in the analysis. The results showed (descriptive statistics are presented in Table 4): regarding the number of role positioning errors per match, t(21) = 19.62, *p* < 0.001; regarding the completeness of tactical execution (assessed by coaches), t(21) = −122.00, *p* < 0.001; and regarding the frequency of effective communication among team members per match, t(21) = −57.12, *p* < 0.001. These results indicate the positive effect of team collaboration psychological training on improving the team's athletic performance, which effectively enhanced the athletes’ team awareness and collaboration ability.

**Table 4:** Descriptive statistics of competition performance indicators before and after training (M ± SD).

Evaluation indicators	Pre-training（8 rounds）	Post-training（8 rounds）
Number of role positioning errors per session	4.2 ± 0.8 times	1.7 ± 0.5 times
Degree of Coach's Tactical Execution	71.3% ± 4.2%	89.6% ± 3.8%
Frequency of effective communication among team members per session	28.5 ± 3.4 times	41.2 ± 4.1 times

## 4 Discussion

Based on Miller's Ice Hockey Success Psychology Model, this study constructed a systematic psychological training system consisting of six modules: goal orientation, emotion regulation, positive imagery, positive self-talk, team collaboration, and injury-resilient psychological intervention (6), and verified the intervention effect of this system through empirical research during the national team training camp period. The results showed that this training system could significantly reduce the level of occupational burnout among Chinese women's 3v3 ice hockey athletes, improve their perceived stress, emotion regulation ability, and attention maintenance ability, and simultaneously significantly enhance performance-related indicators such as athletes’ decision-making accuracy in high-pressure scenarios and team tactical execution efficiency. The hierarchical psychological training program constructed based on this model is compatible with the developmental rules of U10-U18 adolescent ice hockey athletes and is feasible for promotion in the reserve talent training system. The intervention effect of this study is fully consistent with the core theoretical assumptions of the Ice Hockey Success Psychology Model, confirming the positive promoting effect of the multi-module integrated psychological training on ice hockey athletes’ psychological abilities and athletic performance (28).

### 4.1 Analysis of the core psychological mechanisms of intervention effects

The intervention effect of this study is not the improvement of a single psychological skill, but the remodeling of athletes’ psychological regulation patterns from four dimensions (cognition, emotion, behavior, and team) through the synergistic effect of the six modules. The specific mechanisms of action are elaborated as follows:

Mechanism of action of goal-oriented training: The goal-oriented training in this study improved athletes’ self-regulation ability through three core links: clarification of personal motivation, breakdown of preparation goals, and integration of individual-team goals (29). Based on self-determination theory, this training decomposed abstract team competition goals into executable individual phased tasks, and simultaneously deeply bound individual career development with team goals, satisfying the three basic psychological needs of athletes (competence, autonomy, and relatedness), thereby reducing burnout manifestations such as emotional exhaustion and reduced personal accomplishment. This is also the core reason for the significant decrease in the scores of all three dimensions of athletes’ burnout after goal-oriented training in this study (30).

Mechanism of action of emotion regulation training: The 8 week emotion regulation training achieved the improvement of athletes’ emotion regulation ability at both physiological and psychological levels through a stepwise design of “basic breathing regulation - mindfulness awareness - stress decomposition - programmed regulation protocol”. At the physiological level, the 4-7-8 breathing method activates the parasympathetic nervous system by prolonging exhalation time, rapidly reducing athletes’ cortisol levels and heart rate, and alleviating physiological stress responses in high-pressure scenarios. At the psychological level, mindfulness training and three-level stress decomposition exercises help athletes establish non-judgmental awareness of emotions, break the vicious cycle of “stressful events - negative emotions - movement distortion”, enable athletes to quickly focus their attention back on the current task in high-pressure scenarios, thereby improving decision-making accuracy and shortening fatigue recovery time (31).

Mechanism of action of positive imagery training: The 4-week positive imagery training, through a progressive design of “static movement imagery - dynamic movement chain imagery - competition scenario imagery”, optimized movement execution patterns by activating neural pathways in the brain's motor cortex through multi-sensory movement rehearsal based on neuromuscular theory (32). Meanwhile, immersive imagery training of complete competition scenarios (33) requires athletes to maintain sustained attention in multi-cue dynamic scenarios, effectively extending the duration of athletes’ effective attention maintenance and improving tactical prediction accuracy and decision-making speed under multi-task processing, which is consistent with the classic research conclusions in the field of motor imagery (34).

Mechanism of action of positive self-talk training: Studies by Whelan et al. (33) have shown that this module, through the training logic of negative dialogue identification - positive speech reconstruction - situational application, helps athletes identify and correct automatic negative thoughts in competitions based on cognitive-behavioral theory. Through cognitive restructuring, catastrophic and self-denying negative dialogues are replaced with process-focused and positively empowering positive self-talk, which effectively improves athletes’ self-efficacy and reduces the interference of negative thoughts on movement execution and decision-making. This is also the core reason for the improved stability of athletes’ performance in setback and failure scenarios (35).

Mechanism of action of team collaboration psychological training: The 4-week team collaboration training, through four links (role cognition, trust establishment, goal integration, and contingency plan solidification), clarified the role positioning and responsibility boundaries of each athlete in the 3v3 small team based on group dynamics theory (36), established standardized on-court communication norms, and reduced tactical errors caused by role misalignment and poor communication (37). Meanwhile, the in-depth integration of individual and team goals improved team cohesion, aligning athletes’ individual behaviors with team tactical goals, thereby significantly enhancing the completeness of tactical execution and the frequency of effective on-court communication (38).

Mechanism of action of injury-resilient psychological intervention: The injury-related psychological intervention for athletes, through the combination of injury cognitive restructuring, positive imagery and self-talk, helps athletes correct catastrophic cognition of injuries, reduce fear and avoidance behaviors related to injuries, and improve compliance with rehabilitation training, achieving coordinated adjustment of physical and psychological states, and providing psychological support for athletes’ stable training and competition participation (29).

### 4.2 Critical comparison with international similar studies

The core findings of this study are consistent with the conclusions of relevant studies in the field of international sports psychology, while also showing significant differentiated characteristics. In terms of the commonalities of research conclusions, the relieving effect of multi-module psychological training on athletes’ burnout confirmed in this study is consistent with the research conclusions of Colizza et al. (20) on North American professional female ice hockey players, which also found that systematic psychological intervention can significantly reduce the level of emotional exhaustion of female ice hockey players and improve their adaptability to competitions. The promoting effect of emotion regulation training on decision-making ability in high-pressure scenarios in this study is consistent with the meta-analysis conclusions of Laborde et al. (32) on the impact of breathing and mindfulness training on athletes’ cognitive performance, confirming the positive promoting effect of breathing regulation and mindfulness training on elite athletes’ decision-making ability under high pressure. Meanwhile, the intervention effects of positive self-talk and positive imagery training in this study also echo the classic research conclusions of Van Raalte et al. (8) and Williams & Cumming (24), verifying the applicability of these psychological skills in ice hockey.

In terms of the differentiation and innovation of the study, this study makes up for three deficiencies in existing international studies: First, most existing ice hockey psychological intervention studies focus on traditional 11-a-side male ice hockey players, and there is an extreme lack of specialized research on women's 3v3 ice hockey, an emerging Youth Olympic discipline. This study is the first to construct a specialized psychological training system based on the characteristics of women's 3v3 ice hockey, filling the research gap in this sub-field (39). Second, most existing international studies are short-term interventions focusing on a single psychological module, such as separate mindfulness training or self-talk training. In contrast, this study constructs a multi-module integrated intervention system based on the Ice Hockey Success Psychology Model, achieving full-dimensional coverage from individual psychological skills to team dynamics. Compared with single-module intervention, it is more suitable for the team-oriented, high-load, and long-cycle preparation needs of ice hockey (40). Third, most existing studies only focus on intervention for adult elite athletes’ competition preparation, while this study also constructs a hierarchical psychological training system suitable for the U10-U18 age group, transforming the intervention experience from elite competitions into a replicable program for the cultivation of adolescent reserve talents, which is relatively rare in existing international studies on ice hockey psychological training (17).

At the same time, this study also found differences from some international studies: For example, some studies on collective ball sports have shown that general mindfulness training has no significant effect on improving athletes’ on-court decision-making ability, while the emotion regulation training in this study has a significant effect on improving the accuracy of high-pressure decision-making. The core reason is that this study did not adopt a general mindfulness training program, but designed specialized on-ice mindfulness training for the competition scenarios of 3v3 ice hockey, deeply integrating mindfulness practice with athletes’ specialized movements such as gliding, ball touching, and decision-making, which greatly improved the ecological validity of the training. This also provides a new practical idea for mindfulness training in collective ball sports (36).

### 4.3 Scientific contributions and application value of the study

The core scientific contributions of this study are reflected in three aspects: First, it is the first time that the Ice Hockey Success Psychology Model has been applied to the group of Chinese women's 3v3 ice hockey athletes (41), verifying the applicability of this model in non-Western cultural contexts and among Asian female ice hockey athletes, expanding the applicable boundary of the model, and providing an empirical sample from China for global ice hockey psychological training research (42). Second, based on the characteristics of women's 3v3 ice hockey, a multi-module integrated psychological training system was constructed, the mechanism of action of each module was clarified, which fills the gap in existing research on psychological intervention for women's 3v3 ice hockey and provides a standardized empirical framework for psychological training in this discipline (43). Third, a hierarchical psychological training system from adult elite athletes to adolescent reserve talents was established, breaking the limitation of existing studies that “elite preparation is disconnected from adolescent training”, and providing a replicable theoretical and practical framework for the full-cycle cultivation of psychological resilience in ice hockey (44).

At the application level, the psychological training system constructed in this study does not occupy the core technical and tactical training time of athletes, can be directly embedded into the daily training camp and preparation cycle of ice hockey, and has strong operability. Meanwhile, the hierarchical training program can be directly promoted to adolescent ice hockey training camps, providing an implementable path for the cultivation of psychological resilience in reserve talents of China's ice hockey program (38).

### 4.4 Limitations

This study verified the positive intervention effect of the multi-module psychological training system constructed based on the Psychology of Ice Hockey Success Model on the psychological state and athletic performance of Chinese women's 3v3 ice hockey athletes. However, limited by the implementation scenario of the national team training camp, sample source, and research cycle, there are still certain limitations. The training program of this study was customized and developed for the specialized characteristics of women's 3v3 ice hockey and the preparation needs of national-level elite athletes, and its adaptability to traditional ice hockey and athletes of other competitive levels has not been systematically verified, resulting in certain limitations in the generalizability of the research conclusions. Meanwhile, this study only collected immediate assessment data before and after the intervention, with only a 1 month short-term follow-up set for the emotion regulation module, and no longer-term longitudinal follow-up was conducted. Therefore, it is impossible to fully verify the long-term stability of the intervention effect of this psychological training system. In the future, relevant conclusions can be further improved by expanding the sample coverage and conducting long-term follow-up studies, and the model can be verified in different sports.

## Data Availability

The raw data supporting the conclusions of this article will be made available by the authors, without undue reservation.
